# Determinants of taxonomic, functional, and phylogenetic beta diversity in breeding birds within urban remnant woodlots: Implications for conservation

**DOI:** 10.1002/ece3.11426

**Published:** 2024-05-14

**Authors:** Yu Liu, Yun Zhu, Su Wu, Yan Wang, Jie Xie, Kai Zhang, Yu Xu

**Affiliations:** ^1^ School of Life Sciences Guizhou Normal University Guiyang China; ^2^ School of Ecology and Nature Conservation Beijing Forestry University Beijing China

**Keywords:** beta diversity, breeding birds, habitat fragmentation, habitat heterogeneity, nestedness‐resultant component, species turnover

## Abstract

Examining beta diversity of animal assemblages in fragmented habitats, which measures variation in species composition among different fragments, is important for understanding the impact of habitat fragmentation on biodiversity. However, relying solely on taxonomic composition may not provide a comprehensive understanding. Incorporating measures of functional and phylogenetic diversities is essential for elucidating the ecological mechanisms underlying changes in community composition. In addition, prevailing studies often prioritize the evaluation of landscape characteristics within fragments as determinants of beta diversity, neglecting differences in habitat type and plant community composition. In this study, we surveyed birds in 26 remnant woodlot patches (ranging from 0.3 to 290.4 ha) in an urban landscape, southwest China, during the breeding season from 2017 to 2022. We recorded 70 bird species (excluding those recorded only once and high‐flying birds, including raptors, swallows, and swifts), with the number of species per patch varying from 14 to 56. The overall bird taxonomic and phylogenetic beta diversities were primarily contributed by the turnover component, while functional beta diversity was dominated by the nestedness‐resultant component. Patch area and perimeter area ratio significantly influenced the taxonomic, functional, and phylogenetic beta diversities, primarily mediated through the nestedness‐resultant component, while inter‐patch distance had a significant effect via the turnover component. In addition, there was a considerable correlation of bird taxonomic, functional, and phylogenetic beta diversities with habitat type and woody plant beta diversities, including their respective partitioned turnover and nestedness‐resultant components. Our results suggest that bird assemblages in these patches may be regulated by selective extinction, interspecific competition, and environmental filtering. The findings have significant implications for sustainable landscape planning and habitat restoration. Conserving habitat patches of different sizes and maintaining or enhancing habitat heterogeneity between patches can facilitate the persistence of metacommunities.

## Graphical abstract

This study explores the beta diversity of bird assemblages in fragmented urban woodlots in southwest China, emphasizing the importance of considering functional and phylogenetic diversities in addition to taxonomic composition. It underscores the need to account for habitat structure, in addition to landscape characteristics, in beta diversity analyses. The findings provide insights into the regulation of bird assemblages by factors such as selective extinction, interspecific competition, and environmental filtering, offering implications for sustainable landscape planning and habitat restoration.

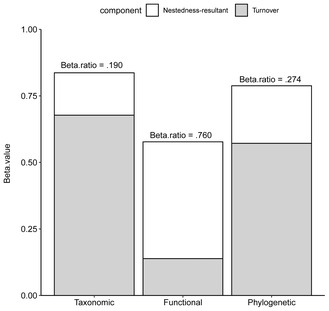

## 1 INTRODUCTION

Habitat fragmentation is the process where large, continuous habitats are divided into many smaller, isolated patches of varying sizes, often caused by natural changes or human activities such as urban construction and agricultural development (Fahrig, 2003). This progress reduces the overall habitat area and increases the isolation between habitat patches, constraining the available living space for species and hindering their dispersal and genetic exchange among populations (Wu, Liang, et al., 2017). These limitations may lead to changes in community structures and ultimately result in a decline or loss of biodiversity (Wilson et al., 2016). Therefore, it is crucial to study changes in species assemblages within fragmented habitats, along with understanding the factors and mechanisms driving these changes. This knowledge is particularly important for formulating effective conservation strategies, especially in urban landscapes where natural habitats are extensively fragmented (Johnson & Munshi‐South, 2017; Miller‐Rushing et al., 2019).

Beta diversity, as conceptualized by Whittaker (1960), captures the variation in species composition diverse communities or environmental gradients (García‐Navas et al., 2020; Gómez‐Rodríguez & Baselga, 2018). Primarily driven by two ecological processes, namely species loss or gain and species turnover, beta diversity is routinely partitioned into nestedness‐resultant and turnover components (Baselga, 2010, 2012). The nestedness‐resultant component refers to differences in species richness or abundance between sites, primarily influenced by selective extinction and colonization dynamics (Si et al., 2015). In the context of habitat fragmentation, smaller patches may initially lose area‐sensitive species, exacerbated by increased human disturbance at patch edges. Consequently, species sensitive to human disturbance might avoid the edges, leading to reduced available area and potential extirpation from patches with a larger perimeter area ratio (PAR), indicative of higher proportion of edge habitat (Wu, Liang, et al., 2017). In addition, increased isolation between habitat patches may pose challenges for species with limited dispersal abilities in navigating more isolated patches, where species richness or abundance are subsets of those found in less isolated patches (Si et al., 2015; Zhao et al., 2021). These factors collectively contribute to the prevalence of the nestedness‐resultant component in beta diversity. Moreover, if different patches demonstrate habitat nestedness, this could also amplify the dominance of the nestedness‐resultant component (Si et al., 2015; Zeng et al., 2022).

On the other hand, the turnover component refers to the process of species replacement in spatial contexts or along environmental gradients (Baselga, 2010, 2012). Key drivers for the turnover component in fragmented habitat include competition, environmental filtering, and geographic barriers (Si, Zhao, et al., 2017). When specific species initially establish themselves in a habitat within a given patch, they may preclude the subsequent colonization of other species through priority effects, resulting in the dominance of species turnover (Chen et al., 2017). In contrast to habitat nestedness, the presence of significant habitat heterogeneity across patches can contribute to an elevated species turnover component, primarily facilitated by environmental filtering (Gutiérrez‐Cánovas et al., 2013; Zhao et al., 2021). In addition, geographic or physical barriers such as mountains or human‐made structures, which prevent species from dispersing, can lead to turnover as different species become isolated in different areas (Leprieur et al., 2011).

Thus, partitioning beta diversity into the nestedness‐resultant and turnover components and establishing their relationships with landscape characteristics and habitat features of patches serve to elucidate how habitat fragmentation affects biodiversity (Si, Zhao, et al., 2017). Nevertheless, an exclusive focus on taxonomic composition of species, as often done in previous studies (e.g., Jesus et al., 2017; Si et al., 2015; Wu, Si, et al., 2017), may prove inadequate. The incorporation of functional and phylogenetic diversities provides a more comprehensive understanding of the underlying ecological processes that drive changes in community composition (Zhao et al., 2021). Phylogenetic diversity integrates information about the evolutionary relationships among species in the community (Webb et al., 2002), while functional diversity is intimately linked to the ecological roles of species, their interactions among themselves, and their interactions with the environment (Basile, 2022; Mayani‐Paras et al., 2023). The partitioning of taxonomic, functional, and phylogenetic beta diversities may not follow identical patterns across fragmented habitats (Si et al., 2016; Zhao et al., 2021). For example, in the fragmented system of the Thousand Island Lake, Si et al. (2016) found that the bird community had a high taxonomic turnover component but a low functional turnover component. In contrast, Zhao et al. (2021) found that ant communities had higher taxonomic and phylogenetic turnover components but a functional nestedness‐resultant component. While species turnover may occur between fragments, there may be a non‐random, ordered loss of specific clades or functional traits across fragments (Matthews et al., 2015; Zhao et al., 2021). Additionally, although many previous studies have frequently examined the effects of landscape characteristics (including area and isolation) of fragments on beta diversity to infer selective extinction and colonization dynamics, few have explored the potential influence of factors concerning differences in habitat type and plant community composition on the beta diversity patterns of animal communities (e.g., Si et al., 2015; Wu, Si, et al., 2017; Zhao et al., 2021). This knowledge gap leaves the underlying mechanisms, such as environmental filtering, largely unexplored.

In this study, we assessed taxonomic, functional, and phylogenetic beta diversities of breeding bird communities on 26 remaining woodlot patches in an urban landscape, southwest China. The main objectives were to explore how landscape characteristics (difference in area, inter‐patch distance, and difference in PAR), as well as differences in habitat type and woody plant community composition among patches, influence beta diversities and their partitioned components in bird communities. By addressing these questions, the study seeks to deepen our understanding of the mechanisms underlying the impacts of habitat fragmentation on biodiversity. This contributes to establishing a scientific foundation for urban development planning and biodiversity conservation.

## 2 MATERIALS AND METHODS

### 2.1 Study area

**FIGURE 1:**
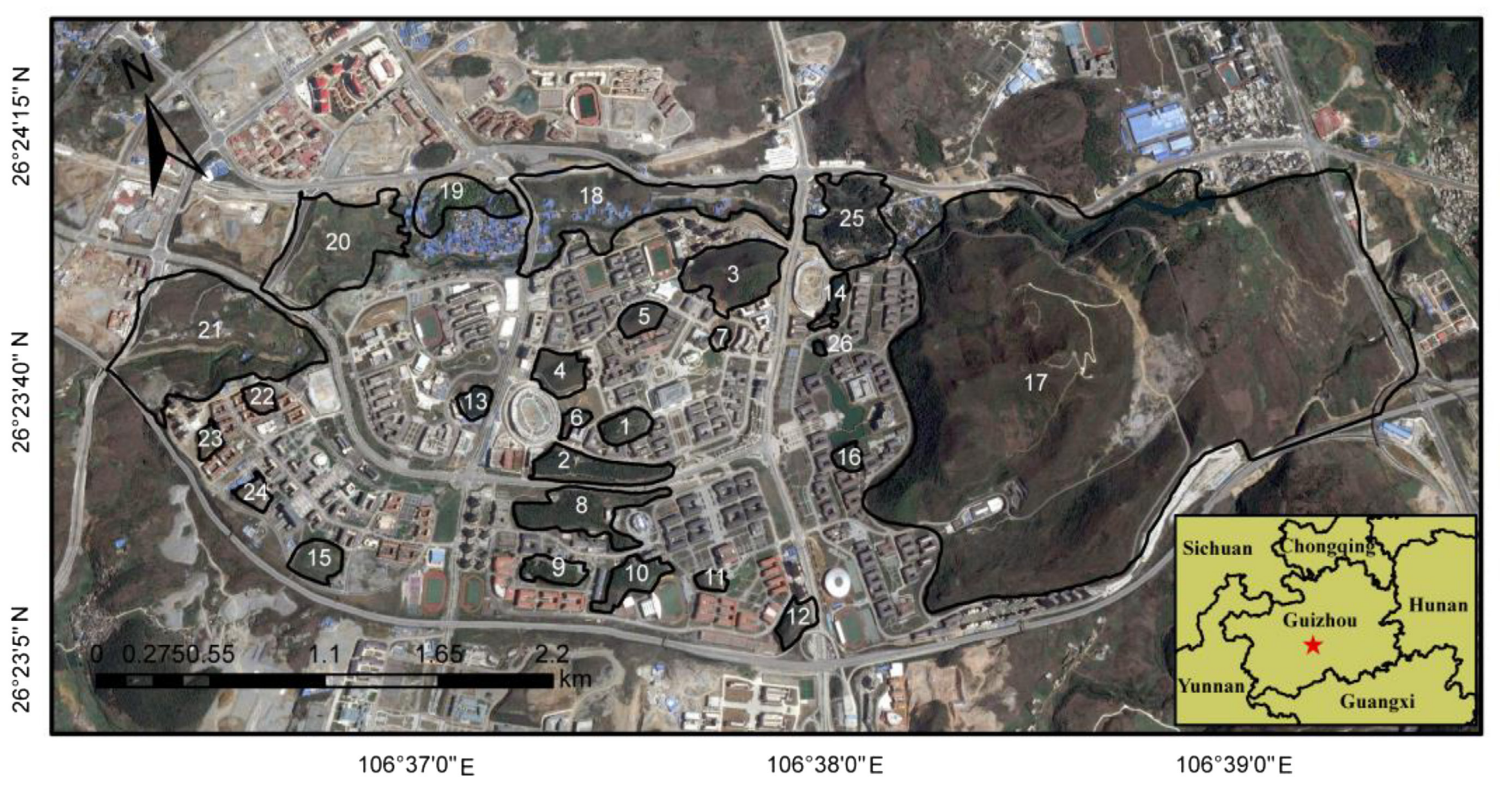
Map showing the location of Huaxi University Town, Guizhou Province, southwest China, and the distribution of 26 woodlot patches overlaid on Google imagery.

This study was carried out in Huaxi University Town (26°22′–26°24′N, 106°36′–106°40′E), Guizhou Province, southwest China (Figure 1). The region features a typical karst landform at an elevation of 1200 m, characterized by a subtropical humid temperate climate with an annual mean temperature of approximately 15.7°C and annual rainfall of about 1070 mm (Zheng et al., 2021). Prior to the construction of the town in 2009, the area comprised natural woodlots surrounded by croplands. The development of the town resulted in fragmentation resulting from the construction of roads and buildings. For our survey, we selected 26 of the remaining woodlots (Figure 1). The habitat in these patches primarily consisted of woodlands, complemented by scrublands, grasslands, wetlands, exposed karst rocks, and croplands. Dominant tree species included *Pinus massoniana*, *Quercus aliena* var. *acutiserrata*, *Platycarya strobilacea*, while shrubs included *Pyracantha fortuneana*, *Coriaria nepalensis*, and *Rosa cymosa* (Cao, 2021). A total of 430 woody plant species have been recorded, with the number of species per patch varying from 25 to 124. The beta diversity of woody plant was predominantly shaped by the turnover component (Cao, 2021).

### 2.2 Field survey and data collection

We acquired high‐resolution satellite images of the study area from Google Earth and delineated the boundary of each patch using ArcGIS 10.7. We measured three landscape matrices of the patches: (1) patch area (ranging from 0.3 to 290.4 ha, with a mean of 17.0 ± 55.3 SD); (2) inter‐patch distance (ranging from 10.8 to 2772.1 m), representing the distance between each pair of patches; (3) and PAR (ranging from 27.0 to 782.7), representing the ratio of the patch's perimeter to its area and serving as a proxy for edge effects.

We surveyed birds using line‐transect methods. In the largest patch (290.4 ha), four transects were established, while in the remaining patches, only one transect was set up each. The length of the transects in each patch ranged from 82.5 to 7073.7 m, roughly proportional to the patch area, aiming to minimize sampling imbalance and error (Schoereder et al., 2004). The survey period extended from 2017 to 2022, covering bird breeding seasons (from April to August) each year. Surveys were usually conducted in favorable weather conditions, excluding rain, wind, or fog. Daily survey times, from 7:00–10:00 and 16:00–18:00, were chosen to coincide with periods of relatively frequent bird activity. During each survey, two observers walked each transect at a constant speed (approximately 1.5 km/h) and recorded birds seen or heard within 50 m along both sides of the transect. To avoid interference from adjacent patches, we ensured that only sounds clearly originating from the surveyed patch were included in our recordings. Bird identification followed the guidelines of MacKinnon et al. (2000) and Viney et al. (2017). A total of 14 rounds of bird surveys were conducted throughout the study: three annually in 2017–2018, four in 2019, one in 2020, two in 2021, and one in 2022.

We selected seven functional traits closely related to birds' resource utilization, reproduction, competition, and dispersal for calculating functional beta diversity, as suggested by Si et al. (2016) and Sinha et al. (2022). These traits included body size, clutch size, trophic level, territoriality, flocking tendency, habitat specificity, and hand‐wing index (HWI; Table S1). For phylogenetic beta diversity, our analyses were based on the global phylogenetic tree of birds from BirdTree (http://birdtree.org) under the option of “Hackett All Species: a set of 10000 trees with 9993 OTUs each” (Jetz et al., 2012). After pruning the global phylogenetic tree to include only the species recorded in our study, we sampled 5000 pseudo‐posterior distributions. Subsequently, we created a Maximum Clade Credibility tree with mean node heights using the BEAST package's TreeAnnonator v1.10.4 (Drummond & Rambaut, 2007). Additionally, we obtained data on habitat types (divided into six categories: woodland, shrubland, grassland, wetland, exposed karst rock, and cropland) and woody plant species from Cao (2021), which used the same transects for bird surveys to assess habitat types and woody plant species in each patch.

### 2.3 Data analyses

We excluded birds recorded only once and high‐flying birds, including raptors, swallows, and swifts, from analysis (Zheng et al., 2021). We calculated the overall bird taxonomic, functional, and phylogenetic beta diversities, as well as the partitioned turnover and nestedness‐resultant components, using the incidence‐based qualitative multiple‐site Sørensen dissimilarity index. For the calculation of functional beta diversity, we initially computed the Gower distance of functional traits. Subsequently, we used principal coordinate analysis (PCoA) for dimensionality reduction and extracted the first three PCoA axes for further analysis. It is noted that, in calculating the overall functional beta diversity, a random selection of 10 patches was performed to prevent computational crashes resulting from an excessive number of patches (Baselga et al., 2023; Villéger et al., 2013; Zhao et al., 2021). We performed 100 resamples to determine their average value. Finally, we used beta.ratio to represent the relative contribution of the nestedness‐resultant component to overall beta diversity. If beta.ratio >.5, it suggests that the beta diversity is primarily contributed by the nestedness‐resultant component; otherwise, it is dominated by the turnover component (Si et al., 2015).

We further used the pairwise‐site Sørensen dissimilarity index to calculate bird taxonomic, functional, and phylogenetic beta diversities between each pair of patches. Then we used multiple regression models (MRMs) based on distance matrices to examine the relationships between the three matrices (bird taxonomic, functional, and phylogenetic beta diversities), including their partitioned components (i.e., nestedness‐resultant and turnover components), and landscape characteristics (difference in area, inter‐patch distance, and difference in PAR). We also calculated habitat type and woody plant beta diversities between each pair of patches using the pairwise‐site Sørensen dissimilarity index. These two matrices, along with their partitioned nestedness‐resultant and turnover components, were also incorporated into the corresponding MRMs, to investigate their relationships with bird beta diversities. The choice of the MRMs was justified by acknowledging that pairwise dissimilarities between patches might be not independent, and the species composition of one patch might affect the dissimilarity of this patch with all others. Furthermore, we used Mantel tests to correct biases in statistical inference and account for potential spatial autocorrelation. Due to a significant correlation between several factors (i.e., difference in area, inter‐patch distance, difference in PAR, habitat type, and woody plant beta diversities; Table S2), we used the partial Mantel test to eliminate the covariation effect caused by the factor with the highest correlation.

Moreover, we conducted an extra analysis by excluding patch 17 (Figure 1), which had a much larger area (290.4 ha) and the smallest PAR (27.0) compared to the other patches, to assess the sensitivity of our results to this potential outlier.

All analyses were performed in R 4.2.2 (R Core Team., 2022) using the packages *betapart* (Baselga et al., 2023), *vegan* (Oksanen et al., 2022), *ecodist* (Goslee & Urban, 2007), *FD* (Laliberté & Legendre, 2010), *dplyr* (Wickham, François, et al., 2023), *tidyr* (Wickham, Vaughan, & Girlich, 2023), and *ggplot2* (Wickham, 2016).

## 3 RESULTS

**FIGURE 2:**
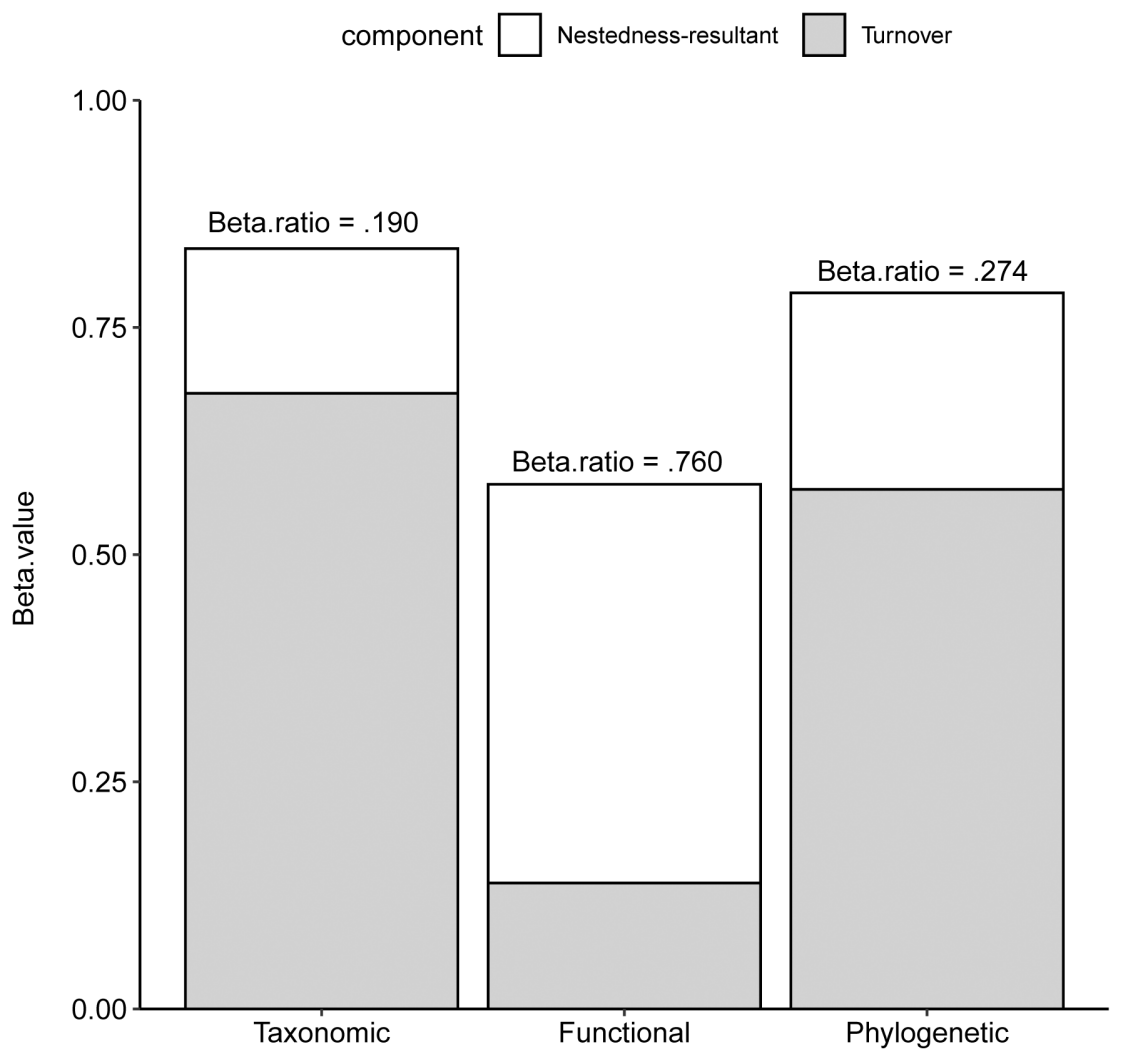
The overall bird beta diversities (including taxonomic, functional, and phylogenetic facets) and their partitioned turnover and nestedness‐resultant components. Beta. ratio indicates the ratio of the nestedness‐resultant component to overall beta diversity.

We recorded 70 bird species across the 26 patches (excluding those recorded only once and high‐flying birds, including raptors, swallows, and swifts), with the number of species per patch varying from 14 to 56. The overall taxonomic (beta.ratio = .190) and phylogenetic (beta.ratio = .274) beta diversities were predominantly contributed by the turnover component, while the functional beta diversity (beta.ratio = .760) was dominated by the nestedness‐resultant component (Figure 2).

**FIGURE 3:**
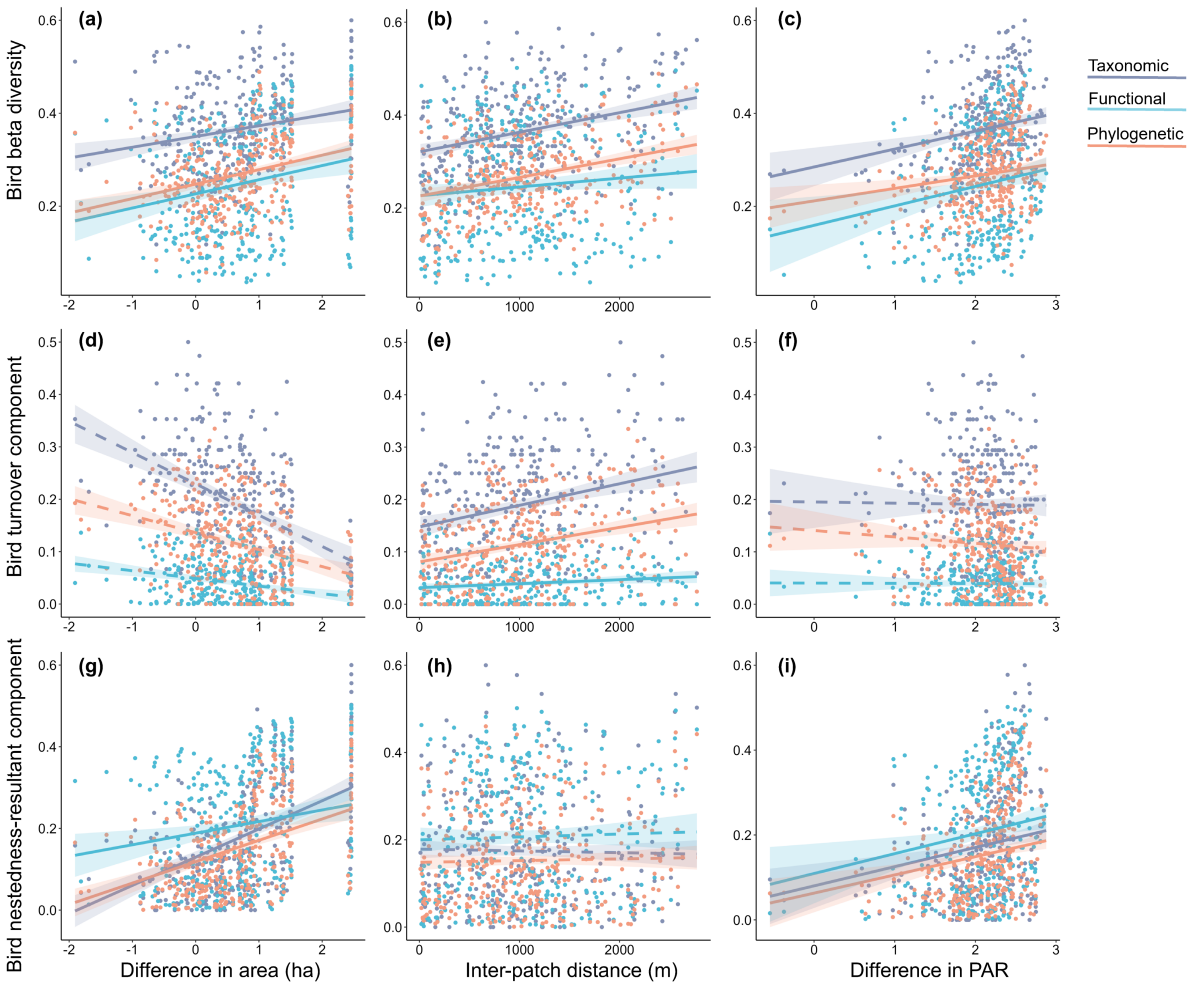
Correlations of bird taxonomic (^T^βsor), functional (^F^βsor), and phylogenetic (^P^βsor) beta diversities with landscape characteristics. (a–c) beta diversity, (d–f) turnover component (^T^βsim, ^F^βsim, ^P^βsim), and (g–i) nestedness‐resultant component (^T^βsne, ^F^βsne, ^P^βsne). Difference in area and difference in PAR were converted to a base‐10 logarithm for analysis. Solid lines indicate statistically significant correlations (*p* < .05), while dashed lines denote non‐significant correlations (*p* > .05); for statistical results, see Table S3.

There were significant positive correlations between the overall bird taxonomic, functional, and phylogenetic beta diversities and differences in patch area and PAR, and inter‐patch distance (Figure 3a–c; Table S3). Concurrently, significant positive correlations were found between the nestedness‐resultant components of the three facets and differences in patch area and PAR; However, no significant correlation was observed with inter‐patch distance (Figure 3g–i). In contrast, the turnover components of the three facets exhibited a significant positive correlation with inter‐patch distance but demonstrated no significant correlation with differences in patch area and PAR (Figure 3d–f).

**FIGURE 4:**
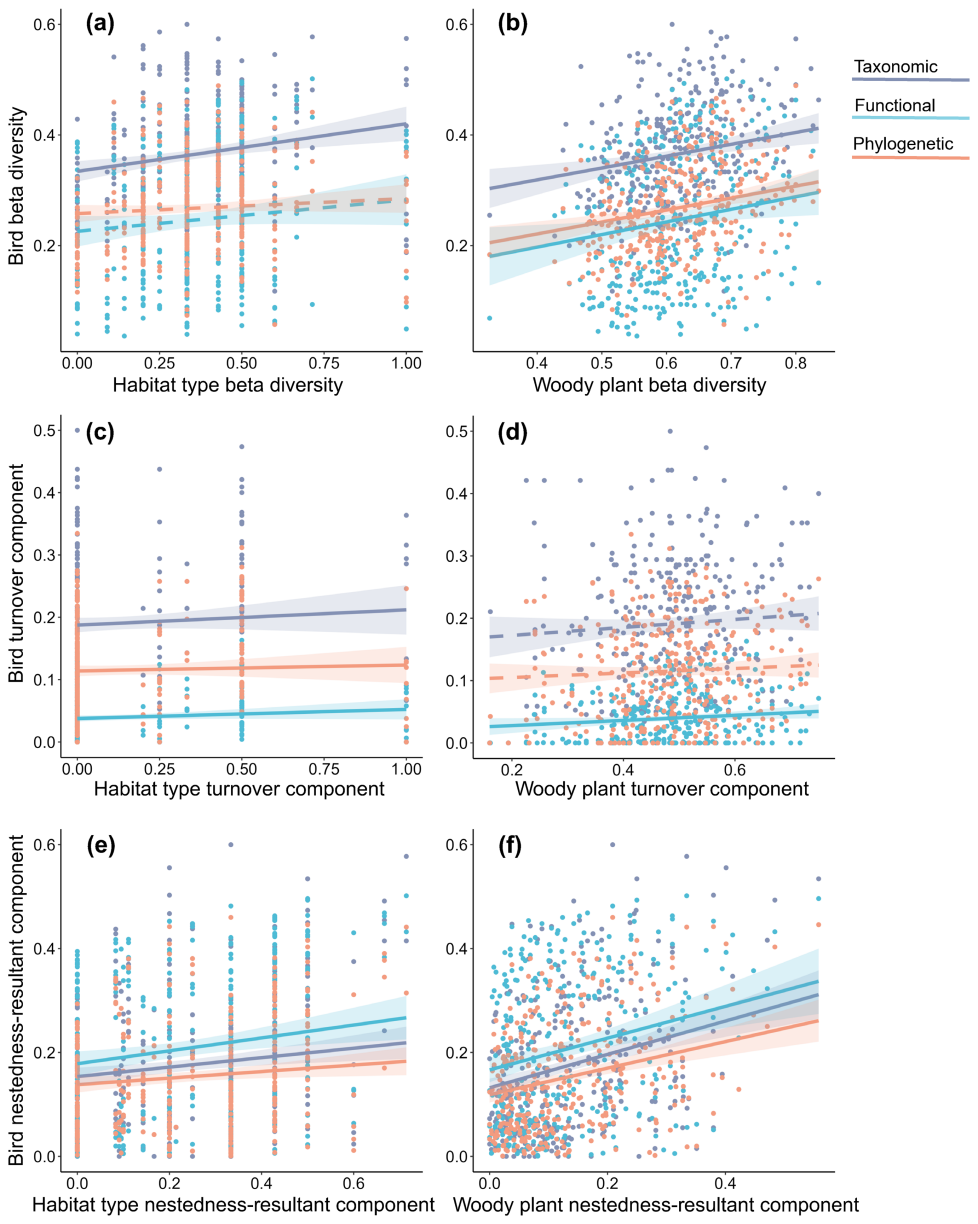
Correlations of bird taxonomic (^T^βsor), functional (^F^βsor), and phylogenetic (^P^βsor) beta diversities with habitat type and woody plant beta diversities. Solid lines indicate statistically significant correlations (*p* < .05), while dashed lines denote non‐significant correlations (*p* > .05); for statistical results, see Table S3.

The overall bird taxonomic, functional, and phylogenetic beta diversities and their turnover and nestedness‐resultant components were all positively correlated with the overall habitat type and woody plant beta diversities and their turnover and nestedness‐resultant components, respectively (Figure 4; Table S3). These relationships were all statistically significant except for the overall bird functional and phylogenetic beta diversities with the overall habitat type beta diversity (Figure 4a), and the bird taxonomic and phylogenetic turnover components with the woody plant turnover component (Figure 4d).

Excluding the patch with a much larger area and the smallest PAR did not significantly impact the overall results (Tables S4 and S5).

## 4 DISCUSSION

The study revealed that the overall bird taxonomic beta diversity in the urban remnant woodlots was primarily influenced by the turnover component, with a relatively low contribution from the nestedness‐resultant component. This pattern aligns with findings in many habitat fragmentation systems (Zeng et al., 2022). Similarly, the overall phylogenetic beta diversity was primarily shaped by the turnover component, indicating that species turnover among lineages with distant phylogenies (Du et al., 2021; Graham & Fine, 2008). In contrast, the overall functional beta diversity was predominantly shaped by the nestedness‐resultant component, as also reported in several other habitat fragmentation systems (e.g., Si et al., 2016; Yang et al., 2022; Zhao et al., 2021). These results suggest that a higher taxonomic (or phylogenetic) turnover does not necessarily correspond to a similarly high functional turnover component.

Habitat fragmentation has the potential to induce the loss of certain species sensitive to environmental changes, but different facets of diversity may respond differently to these changes (Bełcik et al., 2020). Functional diversity, which is closely linked to the ecological niche requirements and interspecific interactions of species, proves more responsive to reductions in habitat area and increases in isolation compared to taxonomic or phylogenetic diversity (Barbaro & Van Halder, 2009; Dias et al., 2015; Suárez‐Castro et al., 2020). The species that remain after fragmentation may become functionally similar as they adapt to the altered conditions, potentially resulting in functional redundancy among communities (Basile, 2022; Ricotta et al., 2020; Tsianou et al., 2021). For example, species like *Phoenicurus leucocephalus*, *Culicicapa ceylonensis*, and *Tachybaptus ruficollis*, despite belonging to different families and being distributed in different patches, manifest comparable functional traits such as trophic level, territoriality, and flocking tendency. Further studies using null models to quantify the functional structure of bird communities can provide insights into the extent of true functional redundancy (Si, Cadotte, et al., 2017; Sinha et al., 2022).

We found a significant positive correlation between the bird nestedness‐resultant components (both the taxonomic, functional, and phylogenetic) and differences in patch area and PAR, even though the taxonomic and phylogenetic components were not the primary drivers of overall beta diversities. The findings align with previous studies on other fragmentation systems, suggesting that the nestedness‐resultant component may be driven by selective extinction (e.g., Si et al., 2015; Zeng et al., 2022). Our study site was originally dominated by natural woodlots surrounded by croplands before urban construction ensued. The subsequent establishment of universities led to the creation of fragmented patches with smaller areas and larger PARs. Species sensitive to area or human activities, such as insectivores or understory birds (Si et al., 2016), were more prone to disappearing from these patches due to a lack of required food or specific habitat (Zheng et al., 2021; Zhu et al., 2022). Species in small patches tended to be those of birds such as *Garrulax sannio* and *Passer montanus*, which are more tolerant to certain environmental conditions. This resulted in differences in species richness between patches and thereby contributing to the nestedness‐resultant components.

However, the bird taxonomic, functional, and phylogenetic nestedness‐resultant components were not affected by inter‐patch distance. Similar results have been reported in other habitat fragmentation systems (e.g., Jesus et al., 2017; Si et al., 2015), indicating no dispersal limitation. The relatively close distance between patches in our study system, with some patches separated only by a road, which, combined with the strong dispersal ability of birds, likely reduces the influence of inter‐patch distance on these components. In contrast, the turnover components were not influenced by differences in patch area and PAR, yet exhibited a significant positive correlation with inter‐patch distance. This differs from previous findings in other habitat fragmentation systems that at smaller spatial scales, where bird beta diversity and its partitioning components were generally unaffected by distance between patches (Jesus et al., 2017; Si et al., 2015). As an explanation, we propose that interspecific competition may contribute to shaping species turnover across different patches (Si, Zhao, et al., 2017). Although birds typically possess strong dispersal abilities, which reduce the likelihood of isolation limiting dispersal, intense interspecific competition during breeding (e.g., for defending territories and nesting sites) may cause them to remain within their home range rather than disperse between different patches (Zheng et al., 2021). For example, we observed that birds such as *Dicrurus macrocercus* and *Urocissa erythroryncha* engaged in the behavior of evicting other species to protect their territories. Consequently, this behavior could contribute to heightened species turnover as the distance between patches increases.

There were significant positive correlations of the bird taxonomic, functional, and phylogenetic nestedness‐resultant components with the habitat type and woody plant nestedness‐resultant components. Meanwhile, the bird taxonomic, functional, and phylogenetic turnover components were significantly positively correlated with the habitat type and woody plant turnover components, even though the positive correlation between the bird taxonomic and phylogenetic turnover components and the woody plant turnover component was not significant. These results suggest that the presence of nestedness in habitats can contribute to an increase in the nestedness‐resultant component of bird beta diversity (Si et al., 2015). However, given the dominance of habitat turnover in our study region, environmental filtering may have driven the heightened species turnover in bird communities (Gutiérrez‐Cánovas et al., 2013). In this study site, being a typical karst region with steep slopes and varied gradient, hydrothermal conditions vary significantly between sites. This leads to pronounced differences in habitat type and the woody plant composition, which may in turn influence each other (Table S2), generating substantial habitat heterogeneity (Cao, 2021). The resulting habitat heterogeneity provides opportunities for species to specialize in particular microhabitats, causing a high turnover of bird species between patches (Li et al., 2022; Zellweger et al., 2017).

## 5 CONCLUSION

Our results revealed that the overall bird taxonomic and phylogenetic beta diversities on the remnant woodlot patches were primarily driven by the turnover component, while functional beta diversity was predominantly shaped by the nestedness‐resultant component. These diversity patterns may be attributed to selective extinction, interspecific competition, and environmental filtering. While acknowledging the small geographical scope of our study, which may limit the generalizability of the results, further research is needed to integrate the intensity of human disturbance for in‐depth analysis. Despite these limitations, our findings provide insights into the understanding of the mechanisms underlying the impacts of habitat fragmentation on biodiversity. These insights are significant for sustainable landscape planning and habitat restoration, underscoring the importance of maintaining habitat integrity and heterogeneity to prevent the homogenization of urban vegetation and biodiversity loss (Vaccaro et al., 2022). Conservation efforts should prioritize larger patches capable of supporting a greater number of coexisting species. However, smaller patches, especially those with high species turnover, are also valuable for sustaining regional community structure (Si et al., 2015; Zhu et al., 2022). Protecting habitat patches of different sizes and enhancing habitat heterogeneity between patches can facilitate the persistence of metacommunities (Fahrig et al., 2022).

## AUTHOR CONTRIBUTIONS

**Yu Liu:** Data curation (equal); formal analysis (lead); investigation (equal); methodology (lead); software (lead); validation (lead); visualization (lead); writing – original draft (lead); writing – review and editing (supporting). **Yun Zhu:** Data curation (equal); formal analysis (supporting); investigation (equal); methodology (supporting). **Su Wu:** Investigation (supporting). **Jie Xie:** Investigation (supporting). **Yan Wang:** Investigation (supporting). **Kai Zhang:** Investigation (supporting); methodology (supporting). **Yu Xu:** Conceptualization (lead); data curation (equal); funding acquisition (lead); methodology (lead); project administration (lead); supervision (lead); writing – review and editing (lead).

## CONFLICT OF INTEREST STATEMENT

None declared.

## Supporting information


Appendix S1.

## Data Availability

The data and R script are openly available in Figshare at https://figshare.com/s/56eb98fd4e98700c0f10.
