# Supplementary material for: Determinants of taxonomic, functional, and phylogenetic beta diversity in breeding birds within urban remnant woodlots: Implications for conservation
Source: Ecol Evol. 2024 May 14;14(5):e11426. doi: 10.1002/ece3.11426 (PMC11091548; doi:10.1002/ece3.11426)
Supplement: Supplementary file 1 — Appendix S1. [file ECE3-14-e11426-s001.docx]

**Supplemental Information**

**Table S1 The description and source for bird functional traits**

| Trait | Scale | Description | Source |
| --- | --- | --- | --- |
| Body size | Continuous | Average individual bird body size (including males and females). | Literature (Zhao 2001) |
| Clutch size | Continuous | The number of eggs produced during a single breeding event. | Literature (Zhao 2001) |
| Trophic level | Categorical | Including three trophic categories: carnivores, herbivores, and omnivores. | Literature (Zhao 2001) |
| Territoriality | Categorical | Exclusive, defended access to habitat and food resources throughout the year, including three levels: strong, weak, and none. | Literature (Tobias et al, 2016; Sheard et al, 2020) |
| Flocking tendency | Categorical | The measurements of bird flocking behavior: strictly social, occasionally social, or strictly solitary. | Our observational data and literature (Zhao 2001) |
| Habitat specificity | Continuous | The sum of the species’ habitat type preference. | Literature (Zhao 2001) |
| HWI | Continuous | Hand-wing index, an estimate of wing shape used as a proxy for flight ability in birds. | Literature (Sheard et al, 2020) |

**Table S2 Correlation matrix among patch area, inter-patch distance, PAR, as well as habitat type and woody plant beta diversities and their turnover and nestedness-resultant components.** Significance levels: **p* < 0.05, ***p* < 0.01, ****p* < 0.001. “\” represents that the correlation was not tested due to no meaningful relationship.

|  | Area | Inter-patch distance | PAR | Habitat type beta diversity | Woody plant beta diversity | Habitat type turnover component | Woody plant turnover component | Habitat type nestedness-resultant | Woody plant nestedness-resultant |
| --- | --- | --- | --- | --- | --- | --- | --- | --- | --- |
| Area |  |  |  |  |  |  |  |  |  |
| Inter-patch distance | -0.017 |  |  |  |  |  |  |  |  |
| PAR | 0.361** | 0.170* |  |  |  |  |  |  |  |
| Habitat type beta diversity | -0.047 | 0.038 | 0.038 |  |  |  |  |  |  |
| Woody plant beta diversity | -0.022 | 0.220** | 0.108 | 0.463*** |  |  |  |  |  |
| Habitat type turnover component | -0.263 | 0.013 | -0.053 | \ | \ |  |  |  |  |
| Woody plant turnover component | -0.229 | -0.032 | -0.023 | \ | \ | 0.256*** |  |  |  |
| Habitat type nestedness-resultant | 0.291*** | 0.025 | 0.111 | \ | \ | \ | \ |  |  |
| Woody plant nestedness-resultant | 0.223* | 0.206** | 0.109 | \ | \ | \ | \ | 0.139* |  |

**Table S3 Results of MRMs and Mantel tests for bird taxonomic, functional, and phylogenetic beta diversities (^T^βsor, ^F^βsor, ^P^βsor), including their turnover (^T^βsim, ^F^βsim, ^P^βsim) and nestedness-resultant (^T^βsne, ^F^βsne, ^P^βsne) components**. These were analyzed for correlations with landscape characteristics (including difference in patch area, inter-patch distance, and difference in PAR), as well as habitat type and woody plant beta diversities (βsor) and their turnover (βsim) and nestedness-resultant (βsne) components.

| Variable | | Bird beta diversity | | MRMs | | | Mantel tests | |
| --- | --- | --- | --- | --- | --- | --- | --- | --- |
|  |  |  |  | Regression slope | Intercept | Standard error | Pearson correlation coefficient | *P*-value |
| Difference in area | | | ^T^βsor | 0.023 | 0.064 | 0.006 | 0.149 | 0.048 |
|  |  |  | ^F^βsor | 0.030 | -0.041 | 0.008 | 0.171 | 0.003 |
|  |  |  | ^P^βsor | 0.031 | 0.007 | 0.005 | 0.267 | 0.004 |
|  |  |  | ^T^βsim | -0.060 | 0.157 | 0.007 | -0.475 | 0.999 |
|  |  |  | ^F^βsim | -0.015 | 0.020 | 0.003 | -0.336 | 0.999 |
|  |  |  | ^P^βsim | -0.032 | 0.109 | 0.005 | -0.373 | 0.999 |
|  |  |  | ^T^βsne | 0.069 | -0.024 | 0.007 | 0.530 | <0.001 |
|  |  |  | ^F^βsne | 0.028 | 0.015 | 0.010 | 0.242 | <0.001 |
|  |  |  | ^P^βsne | 0.052 | -0.023 | 0.006 | 0.480 | <0.001 |
| Inter-patch distance | | | ^T^βsor | 0.000042 | 0.064 | 0.000 | 0.318 | <0.001 |
|  |  |  | ^F^βsor | 0.000019 | -0.041 | 0.000 | 0.115 | 0.043 |
|  |  |  | ^P^βsor | 0.000040 | 0.007 | 0.000 | 0.347 | <0.001 |
|  |  |  | ^T^βsim | 0.000041 | 0.157 | 0.000 | 0.248 | 0.002 |
|  |  |  | ^F^βsim | 0.000008 | 0.020 | 0.000 | 0.122 | 0.042 |
|  |  |  | ^P^βsim | 0.000033 | 0.109 | 0.000 | 0.270 | <0.001 |
|  |  |  | ^T^βsne | -0.000004 | -0.024 | 0.000 | -0.025 | 0.608 |
|  |  |  | ^F^βsne | 0.000007 | 0.015 | 0.000 | 0.043 | 0.192 |
|  |  |  | ^P^βsne | 0.000004 | -0.023 | 0.000 | 0.027 | 0.338 |
| Difference in PAR | | | ^T^βsor | 0.039 | 0.064 | 0.010 | 0.279 | <0.001 |
|  |  |  | ^F^βsor | 0.042 | -0.041 | 0.015 | 0.198 | 0.002 |
|  |  |  | ^P^βsor | 0.027 | 0.007 | 0.008 | 0.255 | 0.001 |
|  |  |  | ^T^βsim | -0.002 | 0.157 | 0.012 | -0.010 | 0.572 |
|  |  |  | ^F^βsim | -0.0004 | 0.020 | 0.005 | -0.005 | 0.534 |
|  |  |  | ^P^βsim | -0.012 | 0.109 | 0.009 | -0.079 | 0.831 |
|  |  |  | ^T^βsne | 0.045 | -0.024 | 0.012 | 0.196 | 0.027 |
|  |  |  | ^F^βsne | 0.047 | 0.015 | 0.017 | 0.164 | 0.010 |
|  |  |  | ^P^βsne | 0.043 | -0.023 | 0.011 | 0.223 | 0.007 |
| Habitat type | βsor | | ^T^βsor | 0.086 | 0.064 | 0.023 | 0.145 | 0.041 |
|  |  |  | ^F^βsor | 0.057 | -0.041 | 0.035 | 0.068 | 0.122 |
|  |  |  | ^P^βsor | 0.027 | 0.007 | 0.020 | 0.025 | 0.391 |
|  | βsim | | ^T^βsim | 0.025 | 0.157 | 0.022 | 0.156 | 0.036 |
|  |  |  | ^F^βsim | 0.015 | 0.020 | 0.009 | 0.153 | 0.018 |
|  |  |  | ^P^βsim | 0.010 | 0.109 | 0.016 | 0.158 | 0.012 |
|  | βsne | | ^T^βsne | 0.091 | -0.024 | 0.030 | 0.179 | 0.013 |
|  |  |  | ^F^βsne | 0.124 | 0.015 | 0.041 | 0.180 | 0.012 |
|  |  |  | ^P^βsne | 0.063 | -0.023 | 0.026 | 0.148 | 0.023 |
| Woody plant | βsor | | ^T^βsor | 0.213 | 0.064 | 0.060 | 0.262 | <0.001 |
|  |  |  | ^F^βsor | 0.228 | -0.041 | 0.090 | 0.175 | 0.004 |
|  |  |  | ^P^βsor | 0.216 | 0.007 | 0.050 | 0.287 | <0.001 |
|  | βsim | | ^T^βsim | 0.063 | 0.157 | 0.049 | 0.131 | 0.098 |
|  |  |  | ^F^βsim | 0.041 | 0.020 | 0.020 | 0.156 | 0.027 |
|  |  |  | ^P^βsim | 0.036 | 0.109 | 0.036 | 0.100 | 0.131 |
|  | βsne | | ^T^βsne | 0.321 | -0.024 | 0.054 | 0.325 | 0.002 |
|  |  |  | ^F^βsne | 0.306 | 0.015 | 0.073 | 0.251 | 0.002 |
|  |  |  | ^P^βsne | 0.255 | -0.023 | 0.047 | 0.310 | 0.002 |

**Table S4 The overall bird beta diversities (including taxonomic, functional, and phylogenetic facets) and their partitioned turnover and nestedness-resultant components (Excluding patch 17).** Beta.ratio indicates the ratio of the nestedness-resultant component to overall beta diversity.

|  | Overall beta diversity | Turnover component | Nestedness-resultant component | Beta.ratio |
| --- | --- | --- | --- | --- |
| Taxonomic | 0.829 | 0.690 | 0.138 | 0.167 |
| Functional | 0.558 | 0.136 | 0.422 | 0.756 |
| Phylogenetic | 0.778 | 0.581 | 0.197 | 0.253 |

**Table S5 Results of MRMs and Mantel tests for bird taxonomic, functional, and phylogenetic beta diversities (^T^βsor, ^F^βsor, ^P^βsor), including their turnover (^T^βsim, ^F^βsim, ^P^βsim) and nestedness-resultant (^T^βsne, ^F^βsne, ^P^βsne) components (Excluding patch 17)**. These were analyzed for correlations with landscape characteristics (including difference in patch area, inter-patch distance, and difference in PAR), as well as habitat type and woody plant beta diversities (βsor) and their turnover (βsim) and nestedness-resultant (βsne) components.

| Variable | | Bird beta diversity | MRMs | | | Mantel tests | |
| --- | --- | --- | --- | --- | --- | --- | --- |
|  |  |  | Regression slope | Intercept | Standard error | Pearson correlation coefficient | *P*-value |
| Difference in area | | ^T^βsor | 0.023 | 0.087 | 0.007 | 0.131 | 0.046 |
|  |  | ^F^βsor | 0.032 | -0.009 | 0.010 | 0.150 | 0.019 |
|  |  | ^P^βsor | 0.036 | 0.029 | 0.006 | 0.236 | 0.015 |
|  |  | ^T^βsim | -0.047 | 0.165 | 0.009 | -0.347 | 0.999 |
|  |  | ^F^βsim | -0.016 | 0.018 | 0.004 | -0.296 | 0.999 |
|  |  | ^P^βsim | -0.027 | 0.111 | 0.007 | -0.283 | 0.997 |
|  |  | ^T^βsne | 0.060 | 0.003 | 0.009 | 0.407 | <0.001 |
|  |  | ^F^βsne | 0.037 | 0.030 | 0.012 | 0.215 | 0.002 |
|  |  | ^P^βsne | 0.055 | -0.002 | 0.008 | 0.409 | <0.001 |
| Inter-patch distance | | ^T^βsor | 0.000048 | 0.087 | 0.000 | 0.350 | <0.001 |
|  |  | ^F^βsor | 0.000018 | -0.009 | 0.000 | 0.105 | 0.049 |
|  |  | ^P^βsor | 0.000043 | 0.029 | 0.000 | 0.359 | <0.001 |
|  |  | ^T^βsim | 0.000049 | 0.165 | 0.000 | 0.322 | <0.001 |
|  |  | ^F^βsim | 0.000008 | 0.018 | 0.000 | 0.151 | 0.017 |
|  |  | ^P^βsim | 0.000036 | 0.111 | 0.000 | 0.313 | <0.001 |
|  |  | ^T^βsne | -0.000003 | 0.003 | 0.000 | -0.023 | 0.611 |
|  |  | ^F^βsne | 0.000008 | 0.030 | 0.000 | 0.044 | 0.193 |
|  |  | ^P^βsne | 0.000006 | -0.002 | 0.000 | 0.036 | 0.286 |
| Difference in PAR | | ^T^βsor | 0.032 | 0.087 | 0.010 | 0.267 | 0.001 |
|  |  | ^F^βsor | 0.038 | -0.009 | 0.015 | 0.189 | 0.005 |
|  |  | ^P^βsor | 0.021 | 0.029 | 0.008 | 0.238 | 0.003 |
|  |  | ^T^βsim | -0.001 | 0.165 | 0.012 | -0.005 | 0.525 |
|  |  | ^F^βsim | 0.001 | 0.018 | 0.005 | 0.013 | 0.432 |
|  |  | ^P^βsim | -0.010 | 0.111 | 0.009 | -0.064 | 0.785 |
|  |  | ^T^βsne | 0.038 | 0.003 | 0.013 | 0.169 | 0.031 |
|  |  | ^F^βsne | 0.041 | 0.030 | 0.017 | 0.147 | 0.017 |
|  |  | ^P^βsne | 0.035 | -0.002 | 0.011 | 0.191 | 0.015 |
| Habitat type | βsor | ^T^βsor | 0.083 | 0.087 | 0.024 | 0.145 | 0.039 |
|  |  | ^F^βsor | 0.044 | -0.009 | 0.035 | 0.068 | 0.125 |
|  |  | ^P^βsor | 0.024 | 0.029 | 0.020 | 0.025 | 0.386 |
|  | βsim | ^T^βsim | 0.029 | 0.165 | 0.023 | 0.156 | 0.037 |
|  |  | ^F^βsim | 0.014 | 0.018 | 0.009 | 0.153 | 0.019 |
|  |  | ^P^βsim | 0.012 | 0.111 | 0.017 | 0.134 | 0.034 |
|  | βsne | ^T^βsne | 0.072 | 0.003 | 0.031 | 0.179 | 0.014 |
|  |  | ^F^βsne | 0.095 | 0.030 | 0.042 | 0.180 | 0.012 |
|  |  | ^P^βsne | 0.047 | -0.002 | 0.027 | 0.148 | 0.023 |
| Woody plant | βsor | ^T^βsor | 0.187 | 0.087 | 0.060 | 0.262 | 0.002 |
|  |  | ^F^βsor | 0.195 | -0.009 | 0.090 | 0.175 | 0.005 |
|  |  | ^P^βsor | 0.193 | 0.029 | 0.050 | 0.288 | 0.001 |
|  | βsim | ^T^βsim | 0.016 | 0.165 | 0.052 | 0.131 | 0.099 |
|  |  | ^F^βsim | 0.040 | 0.018 | 0.022 | 0.156 | 0.026 |
|  |  | ^P^βsim | 0.011 | 0.111 | 0.038 | 0.100 | 0.136 |
|  | βsne | ^T^βsne | 0.257 | 0.003 | 0.058 | 0.325 | 0.001 |
|  |  | ^F^βsne | 0.281 | 0.030 | 0.078 | 0.251 | 0.003 |
|  |  | ^P^βsne | 0.217 | -0.002 | 0.050 | 0.310 | 0.002 |

REFERENCES:

Sheard, C., Neate-Clegg, M. H. C., Alioravainen, N., Jones, S. E. I., Vincent, C., MacGregor, H. E. A., . . . Tobias, J. A. (2020). Ecological drivers of global gradients in avian dispersal inferred from wing morphology. *Nature Communications*, *11*, 2463. https://doi.org/10.1038/s41467-020-16313-6

Tobias, J. A., Sheard, C., Seddon, N., Meade, A., Cotton, A. J., & Nakagawa, S. (2016). Territoriality, social bonds, and the evolution of communal signaling in birds. *Frontiers in Ecology and Evolution*, *4*, 74. https://doi.org/10.3389/fevo.2016.00074

Zhao, Z. (2001). *A Handbook of the Birds of China*. Changchun, Jilin: Jilin Science and Technology Press.
